# Causal relationship between dietary intake and IgA nephropathy: a Mendelian randomization study

**DOI:** 10.3389/fnut.2024.1400907

**Published:** 2024-09-02

**Authors:** Yaping Li, Shengli Wan, Jing Liu, Yilan Huang, Longyang Jiang

**Affiliations:** ^1^Department of Pharmacy, The Affiliated Hospital, Southwest Medical University, Luzhou, China; ^2^Department of Urology, The Affiliated Hospital, Southwest Medical University, Luzhou, China; ^3^School of Pharmacy, Southwest Medical University, Luzhou, China

**Keywords:** dietary intake, IgA nephropathy, Mendelian randomization, genome-wide association study (GWAS), incidence risk

## Abstract

**Objective:**

Previous studies have reported that dietary intake is associated with immunoglobulin A nephropathy (IgAN). However, the causal relationship remains unknown. Based on publicly available genome-wide association study (GWAS) data, we conducted a two-sample Mendelian randomization (MR) analysis to assess the causal association between 26 dietary exposures and IgAN.

**Methods:**

Five methods, including inverse variance weighting (IVW), MR–Egger regression, weighted median, simple mode, and weighted mode, were applied in the MR analysis. To identify the presence of horizontal pleiotropy, we used the MR-Egger intercept test and MR pleiotropy residual sum and outlier (MR-PRESSO) global test. Cochran’s Q statistics were used to assess instrument heterogeneity. We conducted sensitivity analysis using the leave-one-out method.

**Results:**

Finally, the results indicated alcohol intake frequency (odds ratio [OR] (95% confidence interval [CI]) = 1.267 (1.100–1.460), *p* = 0.0010295) was a risk factor of IgAN, while cheese intake (OR (95% CI) = 0.626 (0.492–0.798), *p* = 0.0001559), cereal intake (OR (95% CI) = 0.652 (0.439–0.967), *p* = 0.0334126), and sushi intake (OR (95% CI) = 0.145 (0.021–0.997), *p* = 0.0497) were protective factors of IgAN. No causal relationship was found between IgAN and the rest of the dietary exposures.

**Conclusion:**

Our study provided genetic evidence that alcohol intake frequency was associated with an increased risk of IgAN, while cheese, cereal, and sushi intake were associated with a decreased risk of IgAN. Further investigation is required to confirm these results.

## 1 Introduction

Immunoglobulin A nephropathy (IgAN), a pathological type of chronic kidney disease, is the most common primary glomerular disease worldwide and a leading cause of end-stage renal disease (ESRD) (1). It is characterized by pathological features, including IgA deposition in the glomerular mesangium, glomerular mesangial cell proliferation, and increased mesangial matrices (2). Because IgAN occurs mainly in young and middle-aged people (3), and approximately one-third of IgAN patients irreversibly develop to ESRD within 20 ~ 40 years, it brings a huge economic and social burden (4). Currently, the pathogenesis of IgAN remains unclear. The drug treatment of IgAN relies on renin-angiotensin-aldosterone system inhibitors, glucocorticoids and immunosuppressants (5). However, some patients experience severe adverse drug reactions and poor sensitivity, which makes IgAN treatment challenging. Therefore, it is necessary to conduct a comprehensive search for risk factors related to IgAN and to provide recommendations for the prevention of IgAN.

Previous observational studies have indicated that dietary intake may play an important role in IgAN development. An epidemiological study in Japan found that people who frequently consumed raw eggs and large amounts of carbohydrates had a significantly increased risk of IgAN (6). A retrospective study found that the incidence of IgAN was related to infant milk feeding (7). Kloster Smerud et al. found that food allergies may be related to IgAN (8). It is widely believed that high protein intake (such as red meat, egg, and milk) might lead to increased intraglomerular pressure and glomerular hyperfiltration, which can cause damage to the glomerular structure, leading to or aggravating chronic kidney disease (9). In contrast, plant nutrients and plant-based diets (such as vegetables and fruits) have beneficial effects in patients with chronic kidney disease (10). However, whether the conclusions could be inferred for IgAN patients is unknown, and studies in this area are lacking. Early studies found that alcohol consumption could lead to IgA deposits in the kidneys and increase the incidence risk of IgAN (11, 12); however, some researchers have demonstrated that alcohol intake plays a protective role against chronic kidney disease (13). Two cross-sectional population-based studies conducted in Australia showed that a higher fluid intake appeared to protect the kidney (14). As one of the most popular beverages in the world, tea has been shown to have kidney protective effects (15). However, studies on the association between tea intake and IgAN are lacking. Moreover, owing to inherent defects, existing observational studies cannot efficiently exclude the possibility of reverse causality and confounding factors, which could potentially lead to biased associations and conclusions. Thus, the findings of observational studies need to be clarified further.

In recent years, Mendelian randomization (MR) analysis has been increasingly used to evaluate causal associations between exposure and outcome (16). Unlike conventional observational studies, MR analysis utilizes exposure-related single nucleotide polymorphisms (SNPs) as instrumental variables (IVs) to establish the relationship between risk factors and disease (17). Since genetic variants are randomly assigned during meiosis, MR studies are similar to genetic randomized controlled trials (18), and potential reverse causality and other confounding factors can be efficiently precluded. In the present study, we conducted a two-sample MR analysis to study the causal relationship between the 26 dietary exposures and IgAN.

## 2 Materials and methods

### 2.1 Study design

**Figure 1:**
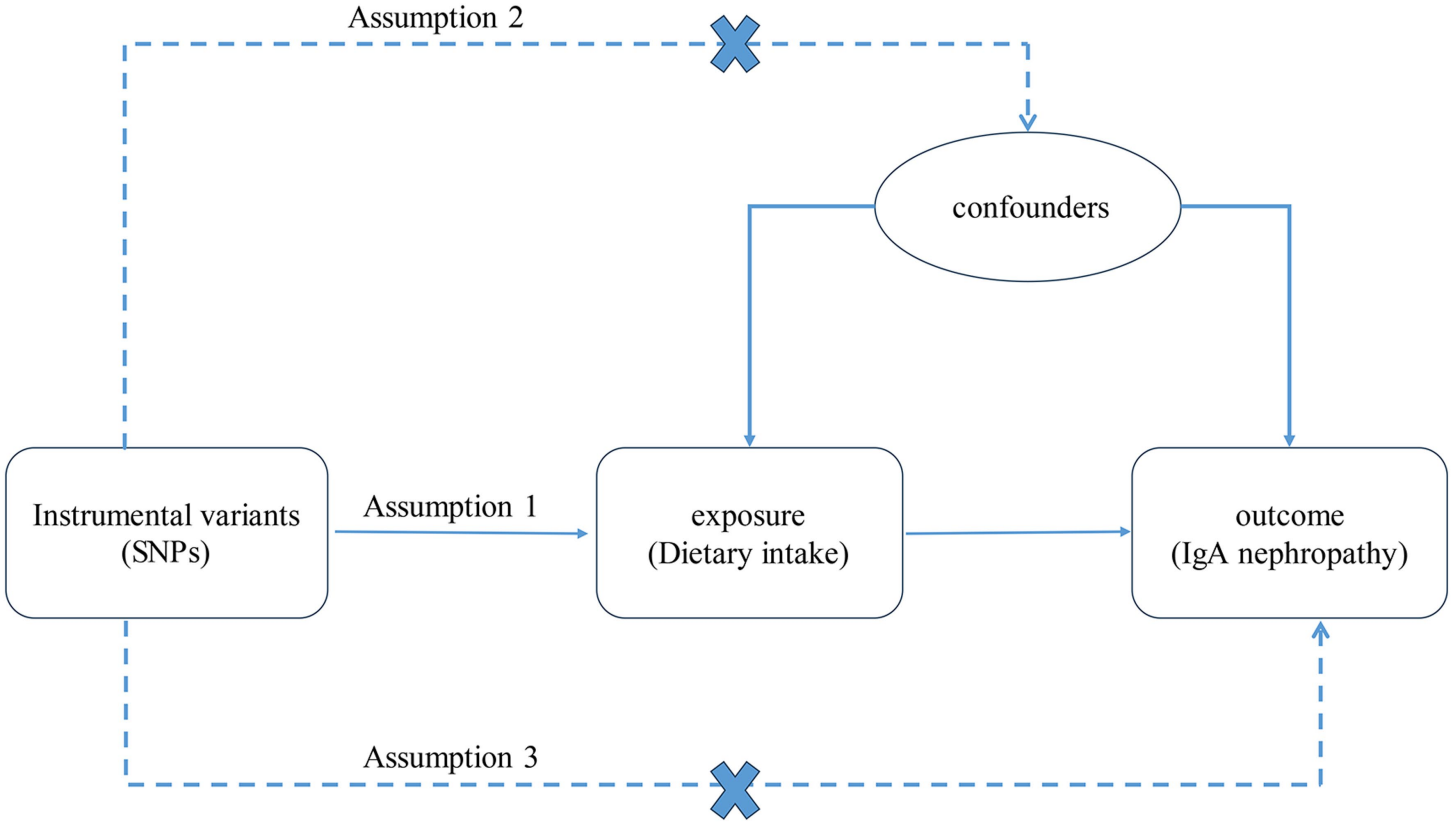
The three assumptions of the MR analysis. SNPs, single nucleotide polymorphisms. IgA, immunoglobulin A.

A two-sample MR analysis was used to evaluate the relationship between dietary intake and IgAN. The selected IVs satisfied three important assumptions (Figure 1): (1) the IVs were strongly related to dietary intake, (2) the IVs were unrelated to any confounding factors, and (3) the IVs had no direct correlation with IgAN via factors other than dietary intake.

### 2.2 Data source

In the present study, exposure data were extracted from the UK Biobank, which collected genotype data on approximately 500,000 individuals. The population included in this study was European. The exposures were mainly categorized as follows: protein, carbohydrates, plant-based diets, and beverages. Finally, the exposures in our study included 26 dietary intakes: cheese (*N* = 451,486), cereal (*N* = 441,640), sushi (*N* = 64,949), alcohol (*N* = 462,346), snackpot (*N* = 64,949), dried fruit (*N* = 421,764), salad/raw vegetable (*N* = 435,435), cooked vegetable (*N* = 448,651), poultry (*N* = 461,900), tofu (*N* = 64,945), Scotch egg (*N* = 64,949), beef (*N* = 461,053), oil fish (*N* = 460,443), non-oil fish (*N* = 460,880), pork (*N* = 460,162), herbal tea (*N* = 64,949), tea (*N* = 447,485), green tea (*N* = 64,949), coffee (*N* = 428,860), mango (*N* = 64,949), pancake (*N* = 64,949), soya dessert (*N* = 64,947), bread (*N* = 452,236), lamb/mutton (*N* = 460,006), processed meat (*N* = 461,981), unsalted peanuts (*N* = 64,949). Outcome data for IgAN (*N* = 477,784, including *N_case_* = 15,587 and *N_control_* = 462,197) were extracted from the EBI database (19). Detailed information is provided in Table 1. As the data were obtained from the IEU Open GWAS Project and could be freely downloaded at https://gwas.mrcieu.ac.uk/, ethical approval and participant consent were not required.

**Table 1:** Detailed information about the aggregated GWAS results.

GWAS ID	Trait	Sample size	SNPs (*n*)	Consortium	Population
ukb-b-1489	Cheese intake	451,486	9,851,867	MRC-IEU	European
ukb-b-15926	Cereal intake	441,640	9,851,867	MRC-IEU	European
ukb-b-5213	Sushi intake	64,949	9,851,867	MRC-IEU	European
ukb-b-5779	Alcohol intake frequency	462,346	9,851,867	MRC-IEU	European
ukb-b-12912	Snackpot intake	64,946	9,851,867	MRC-IEU	European
ukb-b-16576	Dried fruit intake	421,764	9,851,867	MRC-IEU	European
ukb-b-1996	Salad/raw vegetable intake	435,435	9,851,867	MRC-IEU	European
ukb-b-8006	Poultry intake	461,900	9,851,867	MRC-IEU	European
ukb-b-5522	Tofu intake	64,945	9,851,867	MRC-IEU	European
ukb-b-13516	Scotch egg intake	64,949	9,851,867	MRC-IEU	European
ukb-b-2862	Beef intake	461,053	9,851,867	MRC-IEU	European
ukb-b-8089	Cooked vegetable intake	448,651	9,851,867	MRC-IEU	European
ukb-b-2209	Oily fish intake	460,443	9,851,867	MRC-IEU	European
ukb-b-5640	Pork intake	460,162	9,851,867	MRC-IEU	European
ukb-b-13344	Herbal tea intake	64,949	9,851,867	MRC-IEU	European
ukb-b-6218	Mango intake	64,949	9,851,867	MRC-IEU	European
ukb-b-17627	Non-oily fish intake	460,880	9,851,867	MRC-IEU	European
ukb-b-6066	Tea intake	447,485	9,851,867	MRC-IEU	European
ukb-b-5237	Coffee intake	428,860	9,851,867	MRC-IEU	European
ukb-b-4078	Green tea intake	64,949	9,851,867	MRC-IEU	European
ukb-b-6500	Pancake intake	64,949	9,851,867	MRC-IEU	European
ukb-b-998	Soya dessert intake	64,947	9,851,867	MRC-IEU	European
ukb-b-11348	Bread intake	452,236	9,851,867	MRC-IEU	European
ukb-b-14179	Lamb/mutton intake	460,006	9,851,867	MRC-IEU	European
ukb-b-6324	Processed meat intake	461,981	9,851,867	MRC-IEU	European
ukb-b-15555	Unsalted peanuts intake	64,949	9,851,867	MRC-IEU	European
ebi-a-GCST90018866	IgAN	477,784	24,182,646	/	European

GWAS, genome-wide association study; SNPs(n), number of single nucleotide polymorphisms; MRC-IEU, Medical Research Council Integrative Epidemiology Unit; IgAN, immunoglobulin A nephropathy.

### 2.3 Selection of the genetic instruments

To select IVs that fulfilled the three core MR assumptions, we performed a set of quality control techniques. First, SNPs strongly associated with exposure (*p* < 5 × 10^−8^) were selected as IVs. Parameters (*r*^2^ < 0.001 and window size = 10,000) were then set to exclude SNPs with strong linkage disequilibrium (LD). Palindromic structures were excluded. In addition, we calculated F-statistic to quantify the strength of the selected IVs (20) and set a threshold value of *F* > 10 to prevent a weak instrument bias (21). Furthermore, we excluded confounding SNPs by searching for SNP information in wed https://ldlink.nih.gov/?tab=ldtrait, GWAS catalog, and PubMed.

### 2.4 Statistical analysis

The inverse variance weighted (IVW) method was chosen as the primary approach to assess the causal relationship between dietary intake and IgAN. Additional methods, including MR–Egger regression, weighted median, simple mode, and weighted mode were used as complements to the IVW. Cochran’s Q statistics were used to reflect the presence of heterogeneity of instruments (*p* < 0.05 was considered heterogeneity) (22), and horizontal pleiotropy was assessed using the MR-Egger intercept test and MR-PRESSO global test (*p* < 0.05 was considered pleiotropy) (23, 24). We also used the MR-PRESSO method to identify potential outliers. If an outlier was identified, we excluded it and performed the MR analysis again. Finally, we performed a sensitivity analysis of the results using the leave-one-out method. All analyses were performed using the “TwoSampleMR” and “MR-PRESSO” packages in R version 4.3.0.

## 3 Results

### 3.1 Results of SNPs selection and the weak IV test

In our study, we performed an MR analysis on 26 different dietary exposures with IgAN. In the exposure of cheese intake, several SNPs, including rs1024853, rs11649653, rs26579, and rs4776970, were removed for being palindromic with intermediate allele frequencies. For alcohol intake frequency, SNPs including rs1893659 and rs9958320 were removed for incompatible alleles, and rs1104608, rs1894544, and rs62097995 were removed because they were palindromic with intermediate allele frequencies. For exposure of cereal intake, SNPs including rs10837531, rs1104608, rs3859193, rs627185, and rs67723420 were removed because they were palindromic with intermediate allele frequencies. Detailed information of the IVs for cheese intake, alcohol intake frequency, cereal intake, and sushi intake can be found in Supplementary Tables S1–S4. The F statistics of all IVs were greater than 10, which indicates that the results of the MR analysis were not likely to be affected by weak IV bias.

### 3.2 The results of MR analysis

According to Table 2, the primary results of the IVW analysis showed that alcohol intake frequency (OR (95% CI) = 1.267 (1.100–1.460), *p* = 0.0010295) was discovered as a risk factor of IgAN. Cheese intake (OR (95% CI) = 0.626 (0.492–0.798), *p* = 0.0001559), cereal intake (OR (95% CI) = 0.652 (0.439–0.967), *p* = 0.0334126), and sushi intake (OR (95% CI) = 0.145 (0.021–0.997), *p* = 0.049685) were identified as protective factors of IgAN. The rest of the dietary intakes were not related to the occurrence of IgAN (IVW *p* > 0.05).

**Table 2:** The results of IVW about the aggregated GWAS results.

Outcome	Exposure	Method	IVs (*n*)	*b*	se	*p*-value
IgAN	Cheese intake	IVW	53	−0.4677279	0.1236901	0.0001559
IgAN	Alcohol intake frequency^1^	IVW	78	0.236969	0.0721949	0.0010295
IgAN	Cereal intake	IVW	27	−0.4279035	0.2011684	0.0334126
IgAN	Sushi intake	IVW	8	−1.92976	0.983234	0.049685
IgAN	Snackpot intake	IVW	19	2.289872	1.273467	0.072155
IgAN	Dried fruit intake	IVW	41	−0.33966	0.198481	0.087024
IgAN	Salad / raw vegetable intake	IVW	19	1.294252	0.775402	0.09509
IgAN	Poultry intake	IVW	7	−0.69299	0.428516	0.105837
IgAN	Tofu intake	IVW	5	1.744693	1.101154	0.113098
IgAN	Scotch egg intake	IVW	18	1.24625	0.880077	0.156755
IgAN	Beef intake	IVW	14	0.400556	0.290314	0.167668
IgAN	Cooked vegetable intake	IVW	17	0.43797	0.319095	0.169896
IgAN	Oily fish intake	IVW	61	0.209143	0.187539	0.264766
IgAN	Pork intake	IVW	14	0.399173	0.401788	0.32047
IgAN	Herbal tea intake	IVW	19	0.003705	0.00388	0.339667
IgAN	Mango intake	IVW	4	0.276129	0.299107	0.355915
IgAN	Non-oily fish intake	IVW	11	0.467305	0.581542	0.42165
IgAN	Tea intake	IVW	39	−0.08526	0.111566	0.444726
IgAN	Coffee intake	IVW	38	−0.10128	0.169973	0.551258
IgAN	Green tea intake	IVW	21	−0.00108	0.003783	0.775292
IgAN	Pancake intake	IVW	4	−0.24421	0.859624	0.776344
IgAN	Soya dessert intake	IVW	4	0.351673	1.441251	0.807227
IgAN	Bread intake	IVW	30	0.044883	0.25441	0.859963
IgAN	Lamb/mutton intake	IVW	31	0.020207	0.276322	0.941704
IgAN	Processed meat intake	IVW	23	−0.01292	0.21578	0.952263
IgAN	Unsalted peanuts intake	IVW	5	−0.02076	0.909857	0.981796

IVs(n), number of instrumental variables; IgAN, immunoglobulin A nephropathy; IVW, inverse variance weighted. ^1^ The estimates were derived from a random-effects model owing to the presence of heterogeneity based on Cochran’s Q statistic.

The results of the MR–Egger regression, weighted median, simple mode, and weighted mode are presented in Table 3. For alcohol intake frequency (IVW beta = 0.236969, *p* = 0.0010295), similar results were obtained through the MR–Egger regression [beta = 0.0060511, OR (95% CI) = 1.006 (0.729–1.389), *p* = 0.9707445], weighted median [beta = 0.1855399, OR (95% CI) = 1.204 (0.980–1.478), *p* = 0.076532], simple mode [beta = 0.4519185, OR (95% CI) = 1.571 (0.961–2.570), *p* = 0.0757164] and weighted mode [beta = 0.1314467,OR (95% CI) = 1.140 (0.872–1.492), *p* = 0.340832]. For cheese intake (IVW beta = −0.4677279, *p* = 0.0001559), the results of MR–Egger regression [beta = −1.0076467, OR (95% CI) = 1.006 (0.729–1.389), *p* = 0.9707445], weighted median [beta = −0.4766256, OR (95% CI) = 1.204 (0.980–1.478), *p* = 0.076532], simple mode [beta = −0.4767936, OR (95% CI) = 1.571 (0.961–2.570), *p* = 0.0757164] and weighted mode [beta = −0.4767936, OR (95% CI) = 1.140 (0.872–1.492), *p* = 0.340832] were also showed the same trend. For cereal intake (IVW beta = −0.4279035, *p* = 0.0334126) and sushi intake (IVW beta = −1.92976, *p* = 0.049685), the MR-Egger, weighted median, simple mode, and weighted mode analyses also showed consistency.

**Table 3:** Results of the two-sample MR analysis.

Outcome	Exposure	Method	IVs (*n*)	Beta	se	*p*-value	OR (95% CI)
IgAN	Cheese intake	MR Egger	53	−1.0076467	0.5352832	0.065485	0.365 (0.128–1.042)
		Weighted median	53	−0.4766256	0.1653562	0.0039464	0.621 (0.449–0.859)
		IVW	53	−0.4677279	0.1236901	0.0001559	0.626 (0.492–0.798)
		Simple mode	53	−0.4767936	0.3681333	0.200984	0.621 (0.302–1.277)
		Weighted mode	53	−0.4767936	0.3508887	0.1800697	0.621 (0.312–1.235)
IgAN	Alcohol intake frequency	MR Egger	78	0.0060511	0.1644508	0.9707445	1.006 (0.729–1.389)
		Weighted median	78	0.1855399	0.1047554	0.076532	1.204 (0.980–1.478)
		IVW^1^	78	0.236969	0.0721949	0.0010295	1.267 (1.100–1.460)
		Simple mode	78	0.4519185	0.2510124	0.0757164	1.571 (0.961–2.570)
		Weighted mode	78	0.1314467	0.137144	0.340832	1.140 (0.872–1.492)
IgAN	Cereal intake	MR Egger	27	−0.5419623	0.8884959	0.5473818	0.582 (0.102–3.318)
		Weighted median	27	−0.7154268	0.2847579	0.0119912	0.489 (0.280–0.854)
		IVW	27	−0.4279035	0.2011684	0.0334126	0.652 (0.439–0.967)
		Simple mode	27	−1.1549015	0.6413432	0.0833537	0.315 (0.090–1.108)
		Weighted mode	27	−1.0841373	0.526185	0.049501	0.338 (0.121–0.949)
IgAN	Sushi intake	MR Egger	8	−4.04152	5.339624	0.477773	0.018 (0–616.552)
		Weighted median	8	−1.71412	1.211463	0.157094	0.18 (0.017–1.935)
		IVW	8	−1.92976	0.983234	0.049685	0.145 (0.021–0.997)
		Simple mode	8	−1.05946	1.648722	0.540956	0.347 (0.014–8.776)
		Weighted mode	8	−1.17646	1.879474	0.551203	0.308 (0.008–12.272)

IVs(n), number of instrumental variables; IgAN, immunoglobulin A nephropathy; IVW, inverse variance weighted. ^1^ The estimates were derived from a random-effects model owing to the presence of heterogeneity based on Cochran’s Q statistic.

### 3.3 The results of sensitivity analysis

**Figure 2:**
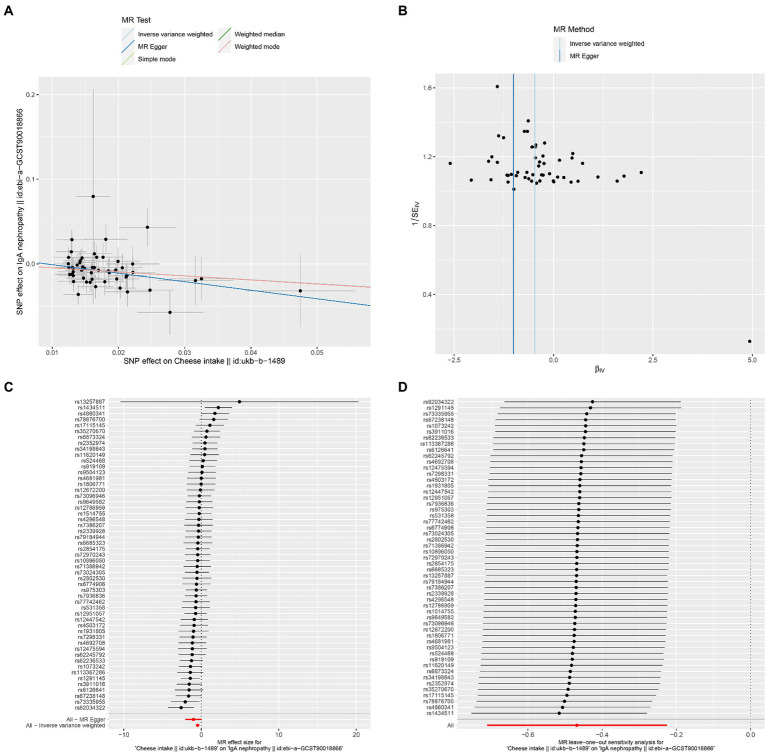
Effects of cheese intake on IgAN. **(A)** Scatter plot of the causal effect of cheese intake on IgAN. **(B)** Funnel plot of the causal effect of cheese intake on IgAN. **(C)** Forest plot of the causal effect of cheese intake on IgAN. **(D)** Forest plot of the leave-one-out analysis.

**Figure 3:**
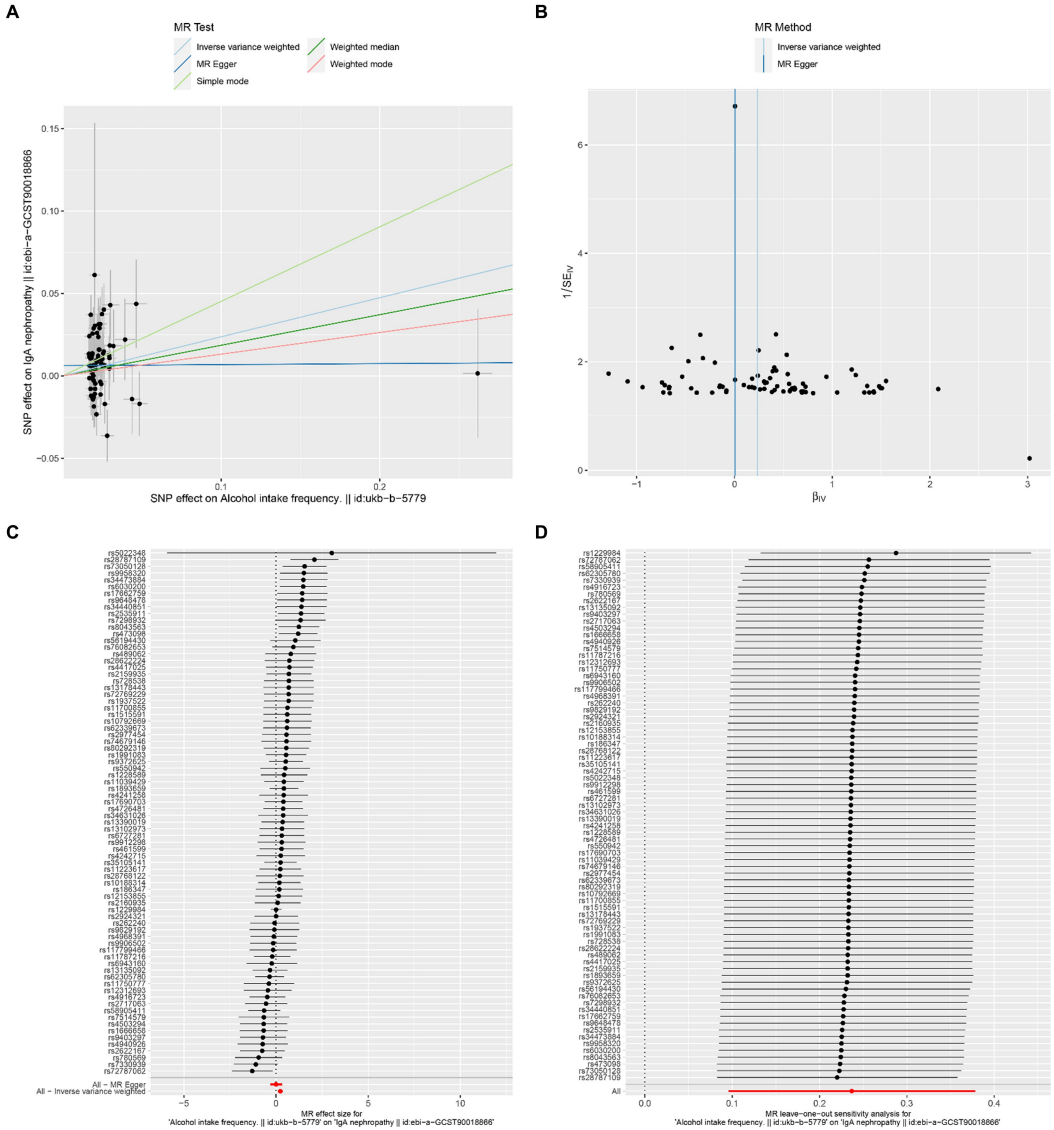
Effects of alcohol intake frequency on IgAN. **(A)** Scatter plot of the causal effect of alcohol intake frequency on IgAN. **(B)** Funnel plot of the causal effect of alcohol intake frequency on IgAN. **(C)** Forest plot of the causal effect of alcohol intake frequency on IgAN. **(D)** Forest plot of the leave-one-out analysis.

**Figure 4:**
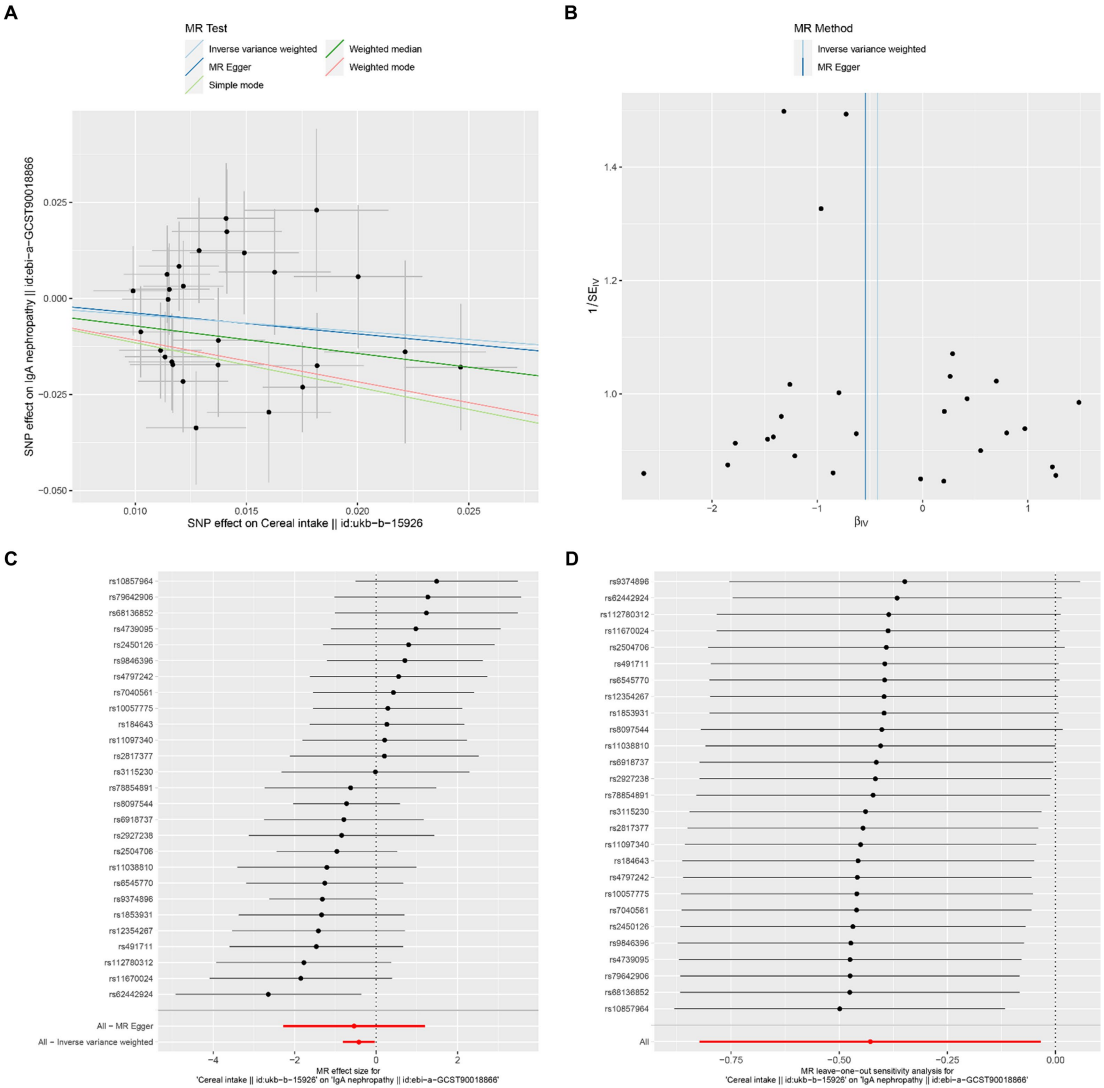
Effects of cereal intake on IgAN. **(A)** Scatter plot of the causal effect of cereal intake on IgAN. **(B)** Funnel plot of the causal effect of cereal intake on IgAN. **(C)** Forest plot of the causal effect of cereal intake on IgAN. **(D)** Forest plot of the leave-one-out analysis.

**Figure 5:**
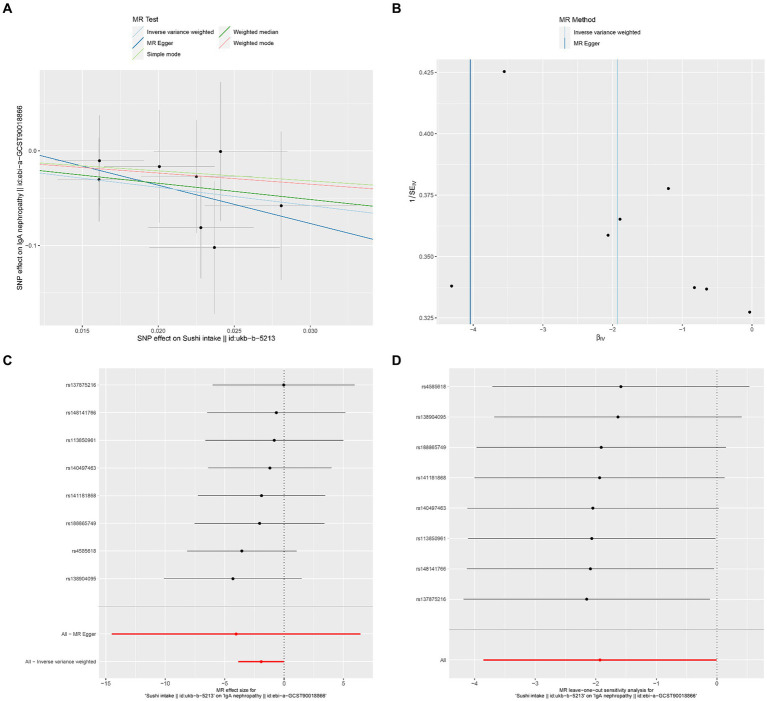
Effects of sushi intake on IgAN. **(A)** Scatter plot of the causal effect of sushi intake on IgAN. **(B)** Funnel plot of the causal effect of sushi intake on IgAN. **(C)** Forest plot of the causal effect of sushi intake on IgAN. **(D)** Forest plot of the leave-one-out analysis.

The results of Cochran’s Q heterogeneity test, MR-Egger intercept test, and MR-PRESSO global test are shown in Table 4. The *p* values of Cochran’s Q for cheese, cereal, and sushi intakes were all greater than 0.05, indicating that there was no heterogeneity. Given *p* < 0.05 of Cochran’s Q test, a random effects IVW MR analysis was used to analysis the relationship between alcohol intake frequency and IgAN (25). There was no evidence of pleiotropy according to the MR-Egger intercept (*p* > 0.05). For alcohol intake frequency, pleiotropy was detected using the MR-PRESSO global test (*p* = 0.047). However, the outlier test did not detect any significant outliers. The leave-one-out method indicated that the results were unaffected after removing each SNP. Scatter plots depict the estimated impact of IVs on exposure and outcome. It is worth mentioning that, rs1229984 is a specific genetic marker for alcohol intake, some researchers used rs1229984 for supplementary sensitivity analysis (26). As reported in the literature (26), we also used the Wald method and conducted an MR analysis using rs1229984 for alcohol intake frequency, but the results were not significant (*p*-value >0.05). However, the results of Mendelian analysis based solely on a single SNP should be considered as a reference, as a polygenic Mendelian randomization investigation would typically have greater power than one including variants from only a single gene region (27). The results of sensitivity analysis indicated that the MR analysis results were reliable (Figures 2–5).

**Table 4:** Reliability test of MR analysis results.

Outcome	Exposure	Method	Cochran’s *Q* test			MR-Egger intercept test *p*-value	MR-PRESSO global test *p*-value
			*Q*	Q_df	*p*-value		
IgAN	Cheese intake	MR Egger	54.346085	51	0.3482498		
		IVW	55.491293	52	0.3445602	0.304777	0.435
	Alcohol intake frequency	MR Egger	97.479231	76	0.0490964		
		IVW	100.59835	77	0.0368427^1^	0.123048	0.047^2^
	Cereal intake	MR Egger	28.683486	25	0.2773632		
		IVW	28.703457	26	0.3247386	0.8960913	0.194
	Sushi intake	MR Egger	1.749363	6	0.941247		
		IVW	1.911264	7	0.964588	0.701355362	0.962

IgAN, immunoglobulin A nephropathy; IVW, inverse variance weighted. ^1^ The Cochran’s Q heterogeneity test *p* < 0.05, a random effects IVW MR analysis was used. ^2^ Although the Global test *p* < 0.05, the outlier test detected no significant outliers.

## 4 Discussion

To the best of our knowledge, this is the first study using the MR method to explore the causal association between dietary intake and risk of IgAN. The results indicated that alcohol intake frequency was associated with a higher risk of IgAN, whereas cheese, cereal, and sushi intake were associated with a lower risk of IgAN.

Previous studies have reported that alcohol consumption might be related to IgAN risk; however, the conclusions showed inconsistent. In 1989, a study found that IgA nephropathy was present in 64% of 107 chronic alcoholics according to samples obtained at forensic autopsy (28). Studies in animal models indicated that alcohol consumption could lead to IgA deposition in the kidneys (12, 29). Further studies on human alcoholics have also found IgA deposits in glomeruli, as well as increased levels of IgA in the circulation (11). However, conclusions drawn from observational studies are not the same. A cross-sectional study including 94 IgAN patients in Japan indicated that alcohol consumption was suggested to have a protective effect against developing IgAN (6). However, this conclusion was not confirmed in a later study by the same group of investigators (30). Another case–control study within 10 years, including 77 patients in China, found that alcohol cessation might have a renal survival benefit in patients with IgAN. However, a cross-sectional study in Finland including 158 patients with IgAN found that moderate alcohol consumption might have a beneficial effect on IgAN (31). The inconsistent conclusions might be due to observational studies’ inability to avoid various confounding factors and the sample size was limited. Based on a large summary of genetic data, our MR study provided genetic evidence that alcohol intake frequency is significantly associated with an increased risk of IgAN. But we cannot evaluate what is the appropriate amount of alcohol consumption, which requires further study.

Few studies have reported the association between cheese intake and IgAN. The protective effects of cheese on IgAN may be explained as follows. First, cheese is a common probiotic food that contains a large amount of live microorganisms. A previous study demonstrated that the disturbance of intestinal microflora, which was characterized by an increase in pathogenic bacteria and a reduction in beneficial bacteria, might play an important role in IgAN (32). The gut-renal connection is an area of new treatment approach for patients with IgAN (33, 34). Lactobacillus and Bifidobacterium are the main members of probiotic bacteria in cheese (35). Studies have found that Lactobacillus had a protective effect against kidney injury (36), and supplementation with probiotics such as Lactobacillus and Bifidobacterium could markedly improve gut dysbiosis and provide significant renal protection in IgAN (37, 38). Second, apart from probiotic bacteria, cheese is also regarded as an antioxidant product that contains various antioxidants, such as casein, lactoferrin, and vitamins C, E, A, and D3 (39). Some studies have demonstrated that oxidative stress and inflammation might play a role in the development and progression of IgAN (40). Moreover, cheese contains various minerals such as calcium, which have an inverse relationship with blood pressure (41) and are beneficial for IgAN patients.

Evidence on the association between cereal intake, sushi intake, and IgAN is also limited. Cereal contains various nutrients, such as proteins, dietary fiber, vitamins, and minerals. A previous study hypothesized that gluten, a kind of protein in cereal, might be involved in the onset of IgAN (42). However, it is worth noting that cereal is also rich in dietary fiber. Some studies demonstrated that dietary fiber could reduce the inflammatory response and modulate gut microbiotas (43, 44). The effects of cereal intake on IgAN should be considered as a function of total nutrient contents, and the mechanism requires further study. Sushi, a type of dish featuring vinegar-flavored rice, is served with other ingredients such as raw or cooked fish strips, vegetables, or seaweed. Although previous studies have found that a large amount of rice intake might increase the risk of IgAN (6, 22), the conclusions drawn from observational studies should be considered carefully. Our study provided genetic evidence that there was a suggestive correlation between sushi intake and a decreased risk of IgAN, but additional studies are still needed to verify our conclusion.

The main advantage of this study is that potential biases, such as confounding factors and reverse causation, were reduced by MR analysis compared with traditionally designed observational studies. Second, our MR analysis was based on the summary data from GWAS with large sample sizes, which diminished weak instrument bias (F-statistic >10). Furthermore, the validity of the results was ensured using multiple sensitivity analyses.

However, our study has several limitations. First, the population included in this study was Europeans. It is unknown whether the current conclusions can be confirmed in non-European populations. Second, we cannot evaluate the nonlinear association between the above exposures and the risk of IgAN. Third, the associations between other dietary factors and the risk of IgAN were not investigated in our study, which is worth to conduct further research. Fourth, given that the IVW *p*-values of sushi intake are approaching 0.05, further validation through additional GWAS data is required in the future.

## 5 Conclusion

Our study indicated that alcohol intake frequency is associated with a significantly increased risk of IgAN. Reducing alcohol intake may be regarded as an important prevention strategy for IgAN. Cheese intake is associated with a significantly reduced risk of IgAN. Moreover, there was a suggestive correlation between cereal intake, sushi intake, and the risk of IgAN. No causal relationship was found between the remaining dietary exposures and IgAN. However, additional studies are required to verify these conclusions.

## Data Availability

The datasets presented in this study can be found in online repositories. The names of the repository/repositories and accession number(s) can be found in the article/Supplementary material.
